# Hydrolysis of phytate and formation of inositol phosphate isomers without or with supplemented phytases in different segments of the digestive tract of broilers

**DOI:** 10.1017/jns.2014.62

**Published:** 2015-01-26

**Authors:** Ellen Zeller, Margit Schollenberger, Imke Kühn, Markus Rodehutscord

**Affiliations:** 1 Institut für Tierernährung, Universität Hohenheim, 70599 Stuttgart, Germany; 2 AB Vista Feed Ingredients, 64293 Darmstadt, Germany

**Keywords:** Inositol phosphate isomers, Phytate hydrolysis, Phytases, Broilers, BD, basal diet, InsP, inositol phosphate, InsP_3_, *myo*-inositol trisphosphate, InsP_4_, *myo*-inositol tetrakisphosphate, InsP_5_, *myo*-inositol pentakisphosphate, InsP_6_, *myo-*inositol 1,2,3,4,5,6-hexakis (dihydrogen phosphate), PhyA, *Aspergillus*-derived phytase Finase^®^ P, PhyE1, *Escherichia coli*-derived phytase Quantum^®^, PhyE2, *E. coli*-derived phytase Quantum^®^ Blue, tP, total P

## Abstract

The objective was to characterise degradation of *myo-*inositol 1,2,3,4,5,6-hexakis (dihydrogen phosphate) (InsP_6_) and formation of inositol phosphate (InsP) isomers in different segments of the broiler digestive tract. Influence of an *Aspergillus niger* (PhyA) and two *Escherichia coli*-derived (PhyE1 and PhyE2) phytases was also investigated. A total of 600 16-d-old broilers were allocated to forty floor pens (ten pens per treatment). Low-P (5·2 g/kg DM) maize–soyabean meal-based diets were fed without (basal diet; BD) or with a phytase added. On day 25, digesta from different digestive tract segments were pooled per segment on a pen-basis, freeze-dried and analysed for P, InsP isomers and the marker TiO_2_. InsP_6_ degradation until the lower ileum (74 %) in BD-fed birds showed a high potential of broilers and their gut microbiota to hydrolyse InsP_6_ in low-P diets. Different InsP patterns in different gut segments suggested the involvement of phosphatases of different origin. Supplemented phytases increased InsP_6_ hydrolysis in the crop (*P* < 0·01) but not in the lower ileum. Measurements in the crop and proventriculus/gizzard confirmed published *in vitro* degradation pathways of 3- and 6-phytases for the first time. In the intestinal segments, specifically formed InsP_4–5_ isomers of supplemented phytases were still present, indicating further activity of these enzymes. *Myo*-inositol tetrakisphosphate (InsP_4_) accumulation differed between PhyE1 and PhyE2 compared with PhyA in the anterior segments of the gut (*P* < 0·01). Thus, the hydrolytic cleavage of the first phosphate group is not the only limiting step in phytate degradation in broilers.

Phytate represents the primary storage form of P in plant seeds. It is defined as any salt of phytic acid (*myo-*inositol 1,2,3,4,5,6-hexakis (dihydrogen phosphate) or InsP_6_). The utilisation of InsP_6_-P depends on InsP_6_ hydrolysis because P absorption occurs mainly as orthophosphate^(^^1^^)^. InsP_6_-hydrolysing enzymes such as phytases (*myo*-inositol hexakisphosphate phosphohydrolases) catalyse the hydrolytic cleavage of InsP_6_ and its salts via several phosphorylated intermediary products (*myo*-inositol pentakis-, tetrakis-, tris-, bis- and monophosphate) down to *myo*-inositol. The International Union of Pure and Applied Chemistry/International Union of Biochemistry differentiates among three types of phytases: 3-phytases (*EC* 3.1.3.8), 4-/6-phytases (*EC* 3.1.3.26) and 5-phytases (*EC* 3.1.3.72), a classification that refers to the initiating position on the inositol ring during *in vitro* InsP_6_ dephosphorylation. The 6-phytases usually originate from plants and initiate hydrolysis at the d-4 (l-6) position of InsP_6_^(^^2^^)^; 3-phytases are usually of microbial origin (starting hydrolysis at the d-3 (l-1) position), such as the fungal *Aspergillus niger* phytase^(^^3^^–^^5^^)^ or the bacterial *Pseudomonas* phytase^(^^2^^)^. However, *Escherichia coli* phytase as an exception was characterised as a 6-phytase (starting hydrolysis at the d-6 (l-4) position)^(^^6^^,^^7^^)^. We are not aware of any study that has investigated whether this *in vitro-*based classification is reflected also in the pathways of InsP_6_ degradation in the more complex and variable environment of the digestive tract of broilers.

InsP_6_-P has long been assumed to be poorly used by avian species because of the lack of sufficient endogenous InsP_6_-hydrolysing enzymes and the denaturation of intrinsic plant phytases in the stomach and during feed manufacture. In broilers, although the activity of endogenous mucosal phytase in the small intestine has been described^(^^8^^–^^10^^)^, its contribution to InsP_6_ hydrolysis has been considered almost negligible. Although some more recent studies have indicated that InsP_6_-P is highly available for broilers^(^^11^^)^, the origin of phytase activity in the digestive tract is controversial. Some authors have suggested that endogenous mucosal phytase of the small intestine is very capable of high InsP_6_ hydrolysis^(^^12^^,^^13^^)^. Others hypothesise that InsP_6_ is hydrolysed by phytases produced by micro-organisms present in the small intestine and particularly in the caeca^(^^14^^,^^15^^)^.

It is not known which positional inositol phosphate (InsP) isomers are formed by different phytases in the digestive tract of avian species. Some studies have described different positional isomers of InsP_6_ degradation products in several segments of the digestive tract in pigs^(^^16^^–^^18^^)^, but the variations in digestive tract physiology and anatomy between pigs and birds (for example, passage rate, digesta viscosity, presence of a crop, variable pH values) caution against assuming that findings in pigs will be similar to that in broilers. One consequence of the uncertainties regarding the availability of InsP_6_-P is that commercial poultry diets are supplemented with mineral P sources. This costly supplementation increases the P concentration in excreta, which may contribute to environmental problems such as eutrophication of surface waters and exhaustion of global raw phosphate resources^(^^19^^)^. An understanding of the rate of degradation of InsP_6_ to the different positional InsP isomers along the digestive tract would enable a more precise alignment of the feed composition to the birds' P requirements and thus increase the likelihood of averting a ‘potential planet phosphate crisis’^(^^20^^)^. Phytases of different origin, varying in their properties, such as pH optimum, proteolytic stability and kinetic efficiency, may differ in effectiveness with transit as the conditions along the digestive tract change. The *in vitro-*determined pH optimum of most phytase supplements is particularly aligned to the conditions of the anterior segments of the digestive tract^(^^21^^)^.

The first objective of the present study therefore was to characterise InsP_6_ hydrolysis and formation of InsP isomers in different segments of the digestive tract of broilers. The second objective was to investigate the InsP_6_ degradation pattern of different phytase additives and their effectiveness in releasing phosphate in broilers and to compare the findings with known *in vitro* properties. In the absence of supplementary phytase, InsP_6_ was hypothesised to be mainly hydrolysed in the posterior intestinal segments of the digestive tract. In contrast, the different phytase supplements were expected to result in greater overall rates of InsP_6_ hydrolysis and to elicit different InsP patterns in the anterior segments.

## Materials and methods

### Experimental diets

The basal diet (BD) was calculated to contain adequate levels of all nutrients according to the recommendations of the Gesellschaft für Ernährungsphysiologie (Society for Nutrition Physiology)^(^^22^^)^ with the exception of Ca and P. It was mainly based on maize and solvent-extracted soyabean meal (Table 1). Ingredients were chosen to obtain low concentrations of total P (tP), high proportions of InsP_6_-P in tP and low intrinsic phytase activity. Concentrations of Ca and tP were calculated to be 7·9 and 5·0 g/kg of DM, respectively, and these levels were confirmed by analyses (Table 1). Titanium dioxide (TiO_2_) was included at a rate of 5 g/kg as the indigestible marker, and the intended Ti concentration was confirmed by analysis.

**Fig. 1.**
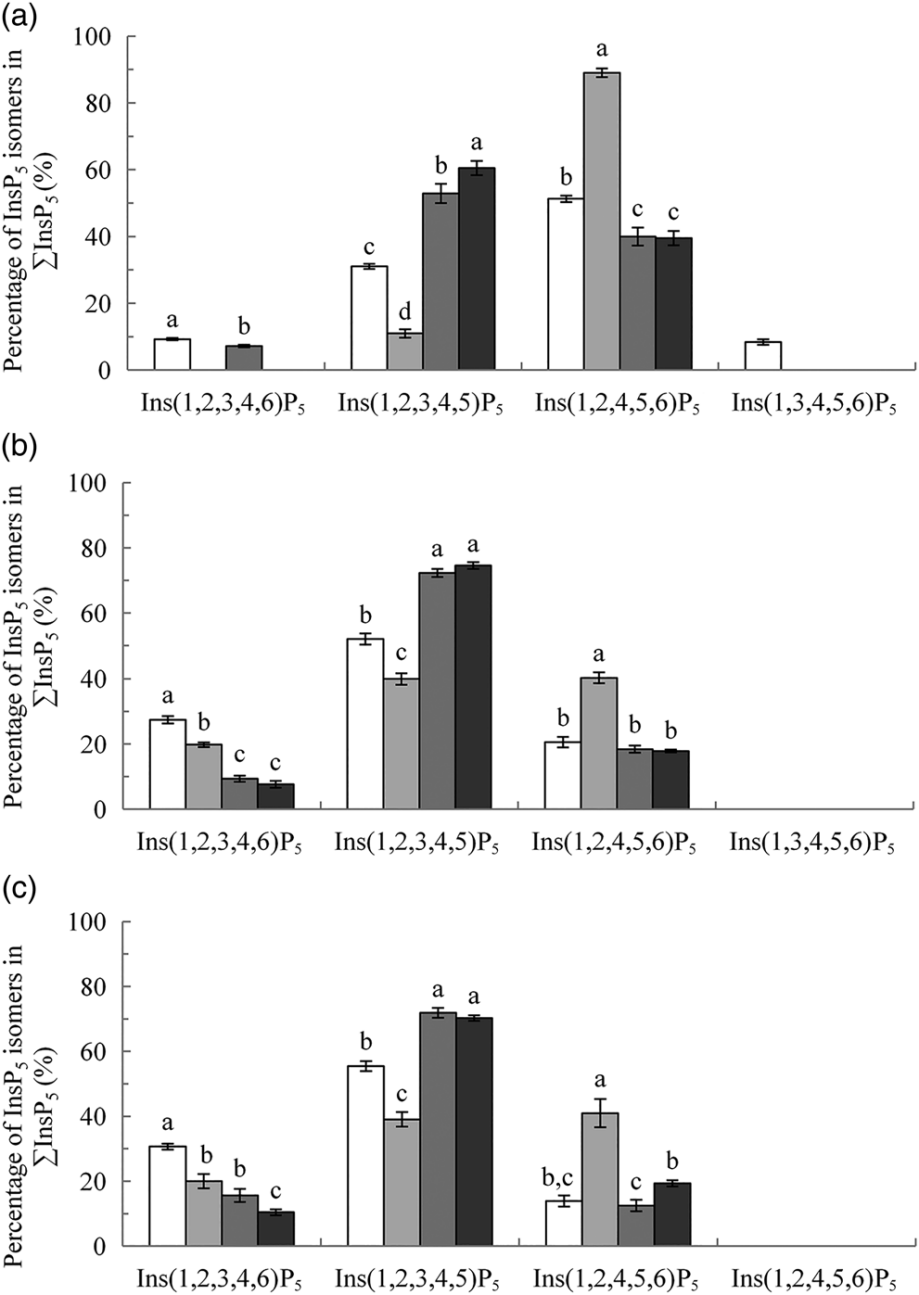
*Myo*-inositol pentakisphosphate (InsP_5_) isomers in the crop (A), duodenum/jejunum (B) and ileum (C), expressed as a percentage of ∑InsP_5_. Values are means, with their standard errors represented by vertical bars. ^a–d^ Values with unlike letters within an InsP_5_ isomer were significantly different (*P* ≤ 0·05; Fisher's protected least significant difference test). Mean separation was only computed if the overall *F* test was significant. □, Basal diet (BD); ░, BD supplemented with *Aspergillus niger* 3-phytase, Finase^®^ P (PhyA); 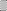, BD supplemented with *Escherichia coli* 6-phytase, Quantum^®^ (PhyE1); 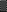, BD supplemented with *E. coli* 6-phytase, Quantum^®^ Blue (PhyE2).

**Table 1.** Ingredient composition and analysed characteristics of the experimental diets

	BD	PhyA	PhyE1	PhyE2
Ingredients (g/kg as fed)				
Maize	553			
Solvent-extracted soyabean meal (48 % crude protein)	400			
Soyabean oil	20			
Limestone	13			
Sodium chloride	1			
Choline chloride	2			
Sodium bicarbonate	3			
Mineral mix*	1			
Vitamin mix†	2			
Titanium dioxide	5			
Analysed characteristics of the diets				
DM (g/kg)	895	896	896	895
Crude ash (g/kg)	53	52	53	51
Crude protein (g/kg)	230	231	232	226
Diethyl ether extract (g/kg)	54	53	52	53
Crude fibre (g/kg)	23	21	24	22
Metabolisable energy (calculated) (MJ/kg)	12·5	12·5	12·5	12·5
Phytase activity (U/kg)‡	<50	399§	467	442
Ca (g/kg DM)	7·5	7·6	7·4	7·3
Total P (g/kg DM)	5·2	5·3	5·2	5·2
InsP_6_-P (g/kg DM)	3·0	3·0	3·0	3·0
Ins(1,2,3,4,6)P_5_ (nmol/g DM)	ND	<LOQ	<LOQ	<LOQ
Ins(1,2,3,4,5)P_5_ (nmol/g DM)	200	200	300	300
Ins(1,2,4,5,6)P_5_ (nmol/g DM)	600	600	600	600
Ins(1,3,4,5,6)P_5_ (nmol/g DM)	ND	ND	ND	ND
InsP_6_ (nmol/g DM)	15 900	16 000	16 200	16 100

BD, basal diet; PhyA, BD supplemented with *Aspergillus niger* 3-phytase, Finase^®^ P; PhyE1, BD supplemented with *Escherichia coli* 6-phytase, Quantum^®^; PhyE2, BD supplemented with *E. coli* 6-phytase, Quantum^®^ Blue; InsP_6_, *myo-*inositol 1,2,3,4,5,6-hexakis (dihydrogen phosphate); InsP_5_, *myo*-inositol pentakisphosphate; ND, not detected; LOQ, limit of quantification.

* P-free: Mineral mix (Gelamin SG 1 Geflügel, GFT MBH), provided per kg of complete diet: 15 mg Cu; 1·6 mg I; 90 mg Fe; 120 mg Mn; 80 mg Zn; 0·5 mg Se; 0·6 mg Co.

† Vitamin mix (Raiffeisen Kraftfutterwerke Süd GmbH), provided per kg of complete diet: 3·6 mg retinol; 75 µg cholecalciferol; 30 mg α-tocopherol; 2·4 mg menadione; 3 mg thiamin; 6 mg riboflavin; 6 mg pyridoxine; 0·03 mg cyanocobalamin; 50 mg nicotinic acid; 14 mg pantothenic acid; 0·1 mg biotin; 1 mg pteroyl(mono)glutamic acid.

‡ Determined at pH 4·5 and 60°C.

§ Determined at pH 5·0 and 37°C.

Diets were prepared in the certified feed mill facilities of Hohenheim University's Agricultural Experiment Station. The BD was mixed in one lot and divided into four equal parts. One part remained without phytase supplementation (BD). The other parts were supplemented with three different phytase-containing products at an intended activity of 500 U/kg of diet. The supplemented phytase products were a commercial *A. niger*-derived 3-phytase (PhyA; Finase^®^ P, *EC* 3.1.3.8; AB Vista) and two *E. coli*-derived thermotolerant 6-phytases (PhyE1 (Quantum^®^) and PhyE2 (Quantum^®^ Blue); *EC* 3.1.3.26; AB Vista). To ensure adequate mixing of each phytase, premixes of each product were prepared by mixing with a small amount of the BD before addition to the treatment diet. Diets were pelleted through a 3-mm die without using steam. The temperature of pellets measured immediately after release from the press ranged between 57°C and 69°C. Representative samples of the diets were taken for analyses of phytase activity, proximate nutrients, DM, Ca, tP, Ti and InsP isomers. The samples were pulverised using a vibrating cup mill (type 6-TOPF; Siebtechnik GmbH) and stored at 4°C until further handling. The experimental diets contained similar concentrations of *myo*-inositol pentakisphosphate (InsP_5_) and InsP_6_ (Table 1). The InsP_6_-P was 57 % of tP in the diets on average and that of InsP_5_-P was 3 % of tP. Lower InsP isomers were not detected in the diets. The phytase activity of the BD was below the limit of detection, but phytase activities of the supplemented diets ranged between 399 and 467 U/kg of diet.

### Animals and management

The study was conducted in the Agricultural Experiment Station of Hohenheim University, location Lindenhöfe in Eningen (Germany). It was approved by the Animal Welfare Commissioner of the University in accordance with the German Welfare Legislation. Birds underwent routine vaccination against coccidiosis, Newcastle disease and infectious bursal disease on 3, 10 and 14 d of age, respectively.

A total of 600 unsexed Ross 308 broilers aged 1 d were obtained from a commercial hatchery (Brüterei Süd GmbH & Co.) and randomly allocated to forty floor pens (approximately 1·5 m × 1·5 m) bedded with wood shavings. Each pen had fifteen birds. The room temperature was 34 and 32°C on days 1 and 2, respectively. Thereafter, the temperature was reduced in steps of 0·5°C per d, reaching 20°C on day 25. Artificial lighting was provided with an intensity of 10 lux. During the first 2 d, the animal house was illuminated continuously. A lighting regimen of 18 h light and 6 h dark was applied from day 3 onwards. Feed and tap water were available for *ad libitum* consumption. Until day 15, the animals were fed a commercial starter diet containing 1·10 % Ca, 0·55 % tP, 22·0 % crude protein, 6·6 % diethyl ether extract and 12·5 MJ metabolisable energy/kg. On day 16, the birds were weighed, and ten pens of fifteen birds were assigned to each of the four dietary treatments and distributed in a completely randomised block design.

### Sampling and analytical methods

At 25 d of age, the animals were asphyxiated by CO_2_ exposure and weighed. To standardise feed intake before sampling and thus retention time of feed in the crop, birds were deprived of feed for 1 h. The feed troughs were then moved back into the pens 1 h before the birds were killed, on an individual-pen basis to ensure the same time schedule for all replicates. The first samples were taken 1 h after the beginning of the light period. Samples from five parts of the digestive tract (crop, proventriculus and gizzard (pooled), duodenum and jejunum (pooled), the terminal part of the ileum (defined as the posterior two-thirds of the section between Meckel's diverticulum and 2 cm anterior to the ileo-caeco-colonic junction^(^^23^^)^) and the caeca) were taken. After opening of the abdominal cavity, the total digestive tract was removed except the crop. Digesta of the intestinal segments were gently flushed out with double-distilled water whereas the segments of the anterior digestive tract (crop, proventriculus and gizzard) were cut open and purged. The samples were pooled for all birds from one pen separately for each segment, immediately frozen at –18°C, freeze-dried (type Delta 1-24; Martin Christ Gefriertrocknungsanlagen GmbH) and pulverised as explained for the diets. The ground samples were stored at 4°C until analysis.

Concentrations of proximate nutrients were determined according to the official methods in Germany (Verband Deutscher Landwirtschaftlicher Untersuchungs- und Forschungsanstalten; VDLUFA)^(^^24^^)^. Feed samples were analysed for DM and crude ash (method 3.1), crude protein (method 4.1.1), diethyl ether extract (method 5.1.1) and crude fibre (method 6.1.1). The concentrations of Ca, tP and Ti in diet and digesta samples were determined by a modification of the method of Boguhn *et al.*^(^^25^^)^. In brief, 20 ml of sulfuric acid (18 mol/l) and 2·5 ml of nitric acid (14 mol/l) were added to 0·4 g of sample. Solutions were heated from 100 to 200°C for 30 min in a block digestion system equipped with a system to trap nitrous gases (Behr K 20 L; Behr Labor-Technik GmbH). After cooling to 100°C, 2·5 ml of the nitric acid were added. Following heating from 225 up to 300°C for 75 min and subsequent cooling to room temperature, the solutions were filled with double-distilled water to a volume of 500 ml and filtered. The Ca, P and Ti concentrations of the solutions were measured using an inductively coupled plasma optical emission spectrometer (VISTA PRO; Varian Inc.) at specific wavelengths for each element according to Shastak *et al.*^(^^26^^)^.

For the analysis of InsP isomers in diet and digesta samples, 1·0 g of the sample was extracted for 30 min with 10 ml of a solution containing 0·2 m-EDTA and 0·1 m-sodium fluoride (pH = 10) as phytase inhibitor using a rotary shaker. The samples were centrifuged at 12 000 ***g*** for 15 min and the supernatant fraction was removed and preserved on ice. The residue was re-suspended in 5 ml of the EDTA–sodium fluoride solution and extracted again for 30 min. The supernatant fractions of the two extraction steps were then combined. A quantity of 1 ml of the pooled supernatant fraction was centrifuged at 14 000 ***g*** for 15 min and 0·5 ml of the resulting supernatant fraction were filtered through a 0·2 µm cellulose acetate filter (VWR) into a Microcon filter (cut-off 30 kDa) device (Millipore) and centrifuged again at 14 000 ***g*** for 30 min. Throughout the whole extraction procedure, the samples were kept below 6°C. The procedure for caecal samples was slightly different: glass beads (diameter 0·6 mm) were added before extraction. To obtain a clear supernatant fraction for the caecal matrix, the extracts were centrifuged for 30 min at 12 000 ***g*** and 6°C. Filtrates were analysed by high-performance ion chromatography and UV detection at 290 nm after post-column derivatisation using an ICS-3000 system (Dionex). InsP with different degrees of phosphorylation (InsP_3–6_) and their positional isomers were separated, without enantiomer differentiation, on a Carbo Pac PA 200 column and corresponding guard column. Fe(NO_3_)_3_ solution (1 g/l, Fe(NO_3_)_3_.9H_2_O, product no. 103883; Merck KGaA) in HClO_4_ (20 g/l, product no. 100518; Merck KGaA) was used as reagent for derivatisation according to Philippy & Bland^(^^27^^)^. The elution order of InsP isomers was established using commercial standards if available. InsP_5_ isomer standards were purchased from Sirius Fine Chemicals. Seven out of nine *myo*-inositol tetrakisphosphate (InsP_4_) and nine out of twelve *myo*-inositol trisphosphate (InsP_3_) isomer standards were available from Santa Cruz Biotechnology. One detected peak out of the group of InsP_4_ isomers could not be attributed but was presumed to be Ins(1,2,4,6)P_4_ by comparison with the elution order of Chen & Li^(^^28^^)^, who used similar chromatographical conditions. A clear identification of the InsP_3_ isomers present was not possible. However, a peak was detected, corresponding in its retention time to the retention time of Ins(1,3,4)P_3_, Ins(1,4,6)P_3_, Ins(1,2,6)P_3_, Ins(1,4,5)P_3_ and Ins(2,4,5)P_3_ (out of the available standards), which all coeluted under the conditions used. InsP_2_ and InsP_1_ could not be analysed with this method. InsP_6_ was used for quantification, and correction factors for differences in detector response for InsP_3–5_ were used according to Skoglund *et al.*^(^^29^^)^. The limit of detection was defined for a signal:noise ratio of 3:1 and was 0·1 µmol/g of DM for InsP_3–4_ isomers and 0·05 µmol/g of DM for InsP_5_ isomers and InsP_6_. The limit of quantification was defined for a signal:noise ratio of 6:1. A mean for an InsP isomer was calculated only if the isomer was detected in at least five out of the ten samples of one treatment. If the detected value was below the limit of quantification in five or more samples, this was noted as less than the limit of quantification in the tables, and means were not calculated. All samples were analysed in duplicate. The InsP concentration is reported on a DM basis.

Because of differences in the extractability of the phytases used, the phytase activity in the diets was determined under product-specific conditions and expressed as U/kg for all diets. Determination of phytase activity was assayed according to the internal, validated methods of the supplier (BD + PhyA: assay at pH 5·5 and 37°C; BD, BD + PhyE1 and BD + PhyE2: assay at pH 4·5 and 60°C). Both assays were run by Enzyme Services & Consultancy.

### Calculations and statistical analysis

Body-weight gain, feed consumption and feed:gain ratio were determined on a pen basis for the period between days 16 and 25. InsP_6_ hydrolysis and P net absorption in the digestive tract (*y*) were calculated for each pen based on the ratio of InsP_6_ or P and Ti according to the generally accepted equation: 
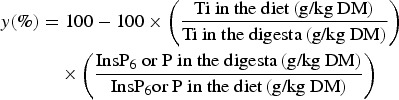


InsP_6_ hydrolysis was calculated for the crop, duodenum/jejunum, lower ileum and caeca. It was not calculated for the proventriculus/gizzard since this segment clearly contained particles of different sizes, which presumably were of variable retention times and thus were not accurately represented by the marker^(^^30^^)^. P net absorption was calculated in the duodenum/jejunum and lower ileum.

The percentage of InsP_3_, ∑InsP_4_ or ∑InsP_5_ in ∑InsP_3–5_ was calculated for each treatment and segment to investigate the rapidity of InsP_6_ hydrolysis and the extent to which intermediary products with different degrees of phosphorylation were formed. Because InsP_1–2_ isomers were not determined, ∑InsP_3–5_ was calculated, representing the sum of identified InsP_6_ hydrolysis products.

Untransformed data are expressed as means with their standard error or the pooled standard error of the mean. Statistical analysis was performed using the MIXED procedure of the software package SAS for Windows (version 9.1.3; SAS Institute Inc.). Before statistical analysis, data that showed non-normal residuals or heterogeneity of variance were log- or square root-transformed. For data expressed as percentages the arc-sine transformation was used. The following statistical model was chosen: y_ij_ = μ + r_i_ + τ_j_ + e_ij_, where y_ij_ is the *i*th measurement in the *j*th treatment, μ is the overall mean, r_i_ is the *i*th block (random), τ_j_ is the effect of the *j*th treatment (fixed) and e_ij_ is the error term. Statistical significance was evaluated by a one-way ANOVA. Mean separation was computed using Fisher's protected least significant difference test (*P* ≤ 0·05) only if the overall *F* test was significant (*P* ≤ 0·05).

## Results

The initial body weight was on average 531 g and was similar between treatments (*P* > 0·05). During the 9-d assay period, average body-weight gain, feed consumption and feed:gain ratio were not significantly different between treatments (Table 2).

**Table 2.** Body-weight (BW) gain, feed consumption (FC) and feed:gain (F:G) ratio of broiler chickens between the ages of 16 and 25 d (Mean values and pooled standard errors; ten pens per treatment with fifteen birds per pen)

	BD	PhyA	PhyE1	PhyE2	Pooled sem
BW gain (g/d)*	59	58	60	56	1·4
FC (g/d)*	95	94	97	92	1·9
F:G ratio (g/g)*	1·61	1·61	1·62	1·63	0·023

BD, basal diet; PhyA, BD supplemented with *Aspergillus niger* 3-phytase, Finase^®^ P; PhyE1, BD supplemented with *Escherichia coli* 6-phytase, Quantum^®^; PhyE2, BD supplemented with *E. coli* 6-phytase, Quantum^®^ Blue.

* The overall *F* test was not significant.

### *Myo*-inositol 1,2,3,4,5,6-hexakis (dihydrogen phosphate) hydrolysis

In birds fed the BD, low InsP_6_ hydrolysis was measured in the crop (9 %; Table 3). Average InsP_6_ hydrolysis in this treatment was 59, 74 and 91 % until the duodenum/jejunum, the lower ileum and the caeca, respectively.

**Table 3.** *Myo-*inositol 1,2,3,4,5,6-hexakis (dihydrogen phosphate) hydrolysis (%) in different segments of the digestive tract of broiler chickens (Mean values and pooled standard errors; ten pens per treatment with fifteen birds per pen)

	BD	PhyA	PhyE1	PhyE2	Pooled sem
Crop	9^d^	64^a^	31^c^	44^b^	4·5
Duodenum/jejunum	59	63	65	68	2·8
Lower ileum	74	74	79	82	3·8
Caeca	91^b^	93^b^	95^a^	96^a^	0·7

BD, basal diet; PhyA, BD supplemented with *Aspergillus niger* 3-phytase, Finase^®^ P; PhyE1, BD supplemented with *Escherichia coli* 6-phytase, Quantum^®^; PhyE2, BD supplemented with *E. coli* 6-phytase, Quantum^®^ Blue.

^a–d^ Mean values in a row with unlike superscript letters were significantly different (*P* ≤ 0·05; Fisher's protected least significant difference test). Mean separation was only computed if the overall *F* test was significant.

Supplementation of phytase had a significant effect on InsP_6_ hydrolysis in the crop (*P* < 0·01) but not in the duodenum/jejunum and lower ileum. In the crop, supplemented phytases significantly increased InsP_6_ hydrolysis, and the effect by PhyA (64 %) was significantly higher than by the other phytase treatments (PhyE1: 31 %; PhyE2: 44 %). Until the duodenum/jejunum and the lower ileum, the difference in InsP_6_ hydrolysis between treatments became lower and statistical difference between treatments disappeared. The average InsP_6_ hydrolysis was 64 % (duodenum/jejunum) and 77 % (lower ileum). InsP_6_ hydrolysis up to the caeca was significantly higher with PhyE1 (95 %) and PhyE2 (96 %) compared with the BD (91 %) and PhyA (93 %) (*P* < 0·01).

### Net absorption of phosphorus

In birds fed the BD, a P net absorption of 34 and 57 % was measured until the duodenum/jejunum and lower ileum (Table 4). Supplementation of phytases caused a significant increase in P net absorption until the duodenum/jejunum (PhyA: 38 %; PhyE1: 38 %; PhyE2: 39 %) (*P* = 0·04). P net absorption until the lower ileum tended to be higher with PhyE1 (60 %) and was significantly higher with PhyE2 (64 %) compared with PhyA (56 %) and BD (57 %) (*P* = 0·03).

**Table 4.** Net absorption of phosphorus (%) in segments of the small intestine of broiler chickens (Mean values and pooled standard errors; ten pens per treatment with fifteen birds per pen)

	BD	PhyA	PhyE1	PhyE2	Pooled sem
Duodenum/jejunum	34^b^	38^a^	38^a^	39^a^	1·2
Lower ileum	57^b^	56^b^	60^a,b^	64^a^	2·1

BD, basal diet; PhyA, BD supplemented with *Aspergillus niger* 3-phytase, Finase^®^ P; PhyE1, BD supplemented with *Escherichia coli* 6-phytase, Quantum^®^; PhyE2, BD supplemented with *E. coli* 6-phytase, Quantum^®^ Blue.

^a,b^ Mean values in a row with unlike superscript letters were significantly different (*P*≤0·05; Fisher's protected least significant difference test). Mean separation was only computed if the overall *F* test was significant.

### Appearance of inositol phosphate isomers

In the crop digesta of birds fed the BD, an average concentration of 638 and 388 nmol/g DM was detected for Ins(1,2,4,5,6)P_5_ and Ins(1,2,3,4,5)P_5_ and lower concentrations were measured for Ins(1,2,3,4,6)P_5_ and Ins(1,3,4,5,6)P_5_ (Table 5 and Fig. 1(A)). The only detectable inositol tetrakisphosphate Ins(1,2,5,6)P_4_ was found in low concentration (141 nmol/g DM), and inositol trisphosphates were not found in the crop in this treatment. About 90 % of ΣInsP_3–5_ in the crop was present as InsP_5_ when the BD was fed. In the proventriculus/gizzard, Ins(1,2,4,5,6)P_5_ and Ins(1,2,3,4,5)P_5_ were the only detectable lower InsP (Table 6), and their concentrations were lower than in the crop. In the duodenum/jejunum, the InsP pattern again was more diverse than in the proventriculus/gizzard and different from the crop (Table 7).

**Table 5.** Concentrations of different inositol phosphate (InsP) isomers (nmol/g DM) in the crop digesta (Mean values and pooled standard errors; ten pens per treatment with fifteen birds per pen)

	BD	PhyA	PhyE1	PhyE2	Pooled sem
InsP_3_*	ND	4268^a^	783^b^	845^b^	214
Proportion of InsP_3_ in ΣInsP_3–5_		0·69^a^	0·15^b^	0·13^b^	0·018
Ins(1,2,4,6)P_4_	ND	ND	ND	ND	
Ins(1,2,3,4)P_4_	ND	ND	170	ND	20·8
Ins(1,2,5,6)P_4_	141^c^	922^c^	2708^b^	4505^a^	421
Proportion of ΣInsP_4_ in ΣInsP_3–5_	0·10^c^	0·14^c^	0·57^b^	0·74^a^	0·032
Ins(1,2,3,4,6)P_5_	116^a^	ND	65^b^	ND	5·1
Ins(1,2,3,4,5)P_5_	388^a,b^	101^c^	475^a^	355^b^	23·0
Ins(1,2,4,5,6)P_5_	638^b^	886^a^	371^c^	241^d^	46·2
Ins(1,3,4,5,6)P_5_	51	ND	ND	ND	8·6
Proportion of ΣInsP_5_ in ΣInsP_3–5_	0·90^a^	0·16^c^	0·28^b^	0·13^c^	0·037
InsP_6_	14 491^a^	5790^d^	10 922^b^	8670^c^	720

BD, basal diet; PhyA, BD supplemented with *Aspergillus niger* 3-phytase, Finase^®^ P; PhyE1, BD supplemented with *Escherichia coli* 6-phytase, Quantum^®^; PhyE2, BD supplemented with *E. coli* 6-phytase, Quantum^®^ Blue; InsP_3_, *myo*-inositol trisphosphate; ND, not detected (the InsP isomer was not detectable in the majority of samples); InsP_4_, *myo*-inositol tetrakisphosphate; InsP_5_, *myo*-inositol pentakisphosphate; InsP_6_, *myo-*inositol 1,2,3,4,5,6-hexakis (dihydrogen phosphate).

^a–d^ Mean values in a row with unlike superscript letters were significantly different (*P* ≤ 0·05; Fisher's protected least significant difference test). Mean separation was only computed if the overall *F* test was significant.

* At least one out of the following InsP_3_ isomers: Ins(1,4,5)P_3,_ Ins(1,2,6)P_3,_ Ins(2,4,5)P_3,_ Ins(1,3,4)P_3,_ Ins(1,4,6)P_3_.

**Table 6.** Concentrations of different inositol phosphate (InsP) isomers (nmol/g DM) in the proventriculus/gizzard digesta (Mean values and pooled standard errors; ten pens per treatment with fifteen birds per pen)

	BD	PhyA	PhyE1	PhyE2	Pooled sem
InsP_3_*	ND	105^c^	348^b^	517^a^	54·2
Proportion of InsP_3_ in ΣInsP_3–5_		0·10	0·09	0·11	0·012
Ins(1,2,4,6)P_4_	ND	ND	ND	ND	
Ins(1,2,3,4)P_4_	ND	ND	<LOQ	ND	
Ins(1,2,5,6)P_4_	ND	254^c^	2457^b^	3537^a^	211
Proportion of ΣInsP_4_ in ΣInsP_3–5_		0·22^c^	0·67^b^	0·79^a^	0·017
Ins(1,2,3,4,6)P_5_	ND	ND	ND	ND	
Ins(1,2,3,4,5)P_5_	92^c^	107^c^	659^a^	295^b^	30·6
Ins(1,2,4,5,6)P_5_	188^b^	618^a^	155^b^	80^c^	28·0
Ins(1,3,4,5,6)P_5_	ND	ND	ND	ND	
Proportion of ΣInsP_5_ in ΣInsP_3–5_	1·00^a^	0·68^b^	0·24^c^	0·09^d^	0·020
InsP_6_	6877^a^	4968^b^	2219^c^	739^d^	196

BD, basal diet; PhyA, BD supplemented with *Aspergillus niger* 3-phytase, Finase^®^ P; PhyE1, BD supplemented with *Escherichia coli* 6-phytase, Quantum^®^; PhyE2, BD supplemented with *E. coli* 6-phytase, Quantum^®^ Blue; InsP_3_, *myo*-inositol trisphosphate; ND, not detected (the InsP isomer was not detectable in the majority of samples); InsP_4_, *myo*-inositol tetrakisphosphate; LOQ, limit of quantification (the InsP isomer was not quantifiable in the majority of samples); InsP_5_, *myo*-inositol pentakisphosphate; InsP_6_, *myo-*inositol 1,2,3,4,5,6-hexakis (dihydrogen phosphate).

^a–d^ Mean values in a row with unlike superscript letters were significantly different (*P* ≤ 0·05; Fisher's protected least significant difference test). Mean separation was only computed if the overall *F* test was significant.

* At least one out of the following InsP_3_ isomers: Ins(1,4,5)P_3,_ Ins(1,2,6)P_3,_ Ins(2,4,5)P_3,_ Ins(1,3,4)P_3,_ Ins(1,4,6)P_3_.

**Table 7.** Concentrations of different inositol phosphate (InsP) isomers (nmol/g DM) in the duodenal/jejunal digesta (Mean values and pooled standard errors; ten pens per treatment with fifteen birds per pen)

	BD	PhyA	PhyE1	PhyE2	Pooled sem
InsP_3_*	ND	ND	ND	ND	
Ins(1,2,4,6)P_4_	ND	ND	ND	ND	
Ins(1,2,3,4)P_4_	355	<LOQ	303	265	75·1
Ins(1,2,5,6)P_4_	ND	<LOQ	123^b^	234^a^	44·9
Proportion of ΣInsP_4_ in ΣInsP_3–5_	0·28		0·26	0·27	0·025
Ins(1,2,3,4,6)P_5_	207^a^	184^a^	110^b^	99^b^	16·7
Ins(1,2,3,4,5)P_5_	399^b^	376^b^	864^a^	957^a^	111
Ins(1,2,4,5,6)P_5_	148^c^	379^a^	214^b,c^	232^b^	31·7
Ins(1,3,4,5,6)P_5_	ND	ND	ND	ND	
Proportion of ΣInsP_5_ in ΣInsP_3–5_	0·72^b^	1·00^a^	0·74^b^	0·73^b^	0·022
InsP_6_	13 392	12 664	11 812	10 701	1076

BD, basal diet; PhyA, BD supplemented with *Aspergillus niger* 3-phytase, Finase^®^ P; PhyE1, BD supplemented with *Escherichia coli* 6-phytase, Quantum^®^; PhyE2, BD supplemented with *E. coli* 6-phytase, Quantum^®^ Blue; InsP_3_, *myo*-inositol trisphosphate; ND, not detected (the InsP isomer was not detectable in the majority of samples); InsP_4_, *myo*-inositol tetrakisphosphate; LOQ, limit of quantification (the InsP isomer was not quantifiable in the majority of samples); InsP_5_, *myo*-inositol pentakisphosphate; InsP_6_, *myo-*inositol 1,2,3,4,5,6-hexakis (dihydrogen phosphate).

^a,b,c^ Mean values in a row with unlike superscript letters were significantly different (*P* ≤ 0·05; Fisher's protected least significant difference test). Mean separation was only computed if the overall *F* test was significant.

* At least one out of the following InsP_3_ isomers: Ins(1,4,5)P_3,_ Ins(1,2,6)P_3,_ Ins(2,4,5)P_3,_ Ins(1,3,4)P_3,_ Ins(1,4,6)P_3_.

The predominant InsP_5_ isomer changed in the intestinal segments (Fig. 1 (B) and (C)). Ins(1,2,3,4,5)P_5_ was the predominant InsP_5_ isomer in the duodenum/jejunum and the subsequent intestinal segments, accompanied by Ins(1,2,3,4,6)P_5_ and Ins(1,2,4,5,6)P_5_ (Tables 7–9). In the duodenum/jejunum, high concentrations of Ins(1,2,3,4)P_4_ (355 nmol/g DM) were noted and it remained the predominant InsP_4_ isomer in subsequent segments in the BD treatment. In the lower ileum, the same pattern of InsP_5_ isomers as in the duodenum/jejunum was found whereas within the InsP_4_ isomers, Ins(1,2,4,6)P_4_ also appeared (Table 8). The pattern of the InsP_5_ isomers in the caeca of birds fed the BD was similar to that of the lower ileum, except that Ins(1,3,4,5,6)P_5_ appeared in relatively low concentrations (75 nmol/g DM) (Table 9). High concentrations of Ins(1,2,3,4)P_4_ (750 nmol/g DM) were detected in the caeca whereas Ins(1,2,4,6)P_4_ was not found and InsP_3_ and traces of Ins(1,2,5,6)P_4_ appeared.

**Table 8.** Concentrations of different inositol phosphate (InsP) isomers (nmol/g DM) in the digesta of the lower ileum (Mean values and pooled standard errors; ten pens per treatment with fifteen birds per pen)

	BD	PhyA	PhyE1	PhyE2	Pooled sem
InsP_3_*	ND	ND	ND	ND	
Ins(1,2,4,6)P_4_	121	ND	<LOQ	<LOQ	30·5
Ins(1,2,3,4)P_4_	309	248	282	216	69·3
Ins(1,2,5,6)P_4_	ND	275	177	192	61·4
Proportion of ΣInsP_4_ in ΣInsP_3–5_	0·34	0·31	0·31	0·31	0·036
Ins(1,2,3,4,6)P_5_	231^a^	209^a^	149^a,b^	106^b^	36·0
Ins(1,2,3,4,5)P_5_	422	410	776	735	156
Ins(1,2,4,5,6)P_5_	110^b^	463^a^	163^b^	195^b^	51·5
Ins(1,3,4,5,6)P_5_	ND	ND	ND	ND	
Proportion of ΣInsP_5_ in ΣInsP_3–5_	0·66	0·69	0·69	0·69	0·036
InsP_6_	11 575	12 348	9965	8913	2089

BD, basal diet; PhyA, BD supplemented with *Aspergillus niger* 3-phytase, Finase^®^ P; PhyE1, BD supplemented with *Escherichia coli* 6-phytase, Quantum^®^; PhyE2, BD supplemented with *E. coli* 6-phytase, Quantum^®^ Blue; InsP_3_, *myo*-inositol trisphosphate; ND, not detected (the InsP isomer was not detectable in the majority of samples); InsP_4_, *myo*-inositol tetrakisphosphate; LOQ, limit of quantification (the InsP isomer was not quantifiable in the majority of samples); InsP_5_, *myo*-inositol pentakisphosphate; InsP_6_, *myo-*inositol 1,2,3,4,5,6-hexakis (dihydrogen phosphate).

^a,b^ Mean values in a row with unlike superscript letters were significantly different (*P* ≤ 0·05; Fisher's protected least significant difference test). Mean separation was only computed if the overall *F* test was significant.

* At least one out of the following InsP_3_ isomers: Ins(1,4,5)P_3,_ Ins(1,2,6)P_3,_ Ins(2,4,5)P_3,_ Ins(1,3,4)P_3,_ Ins(1,4,6)P_3_.

**Table 9.** Concentrations of different inositol phosphate (InsP) isomers (nmol/g DM) in the caecal digesta (Mean values and pooled standard errors; ten pens per treatment with fifteen birds per pen)

	BD	PhyA	PhyE1	PhyE2	Pooled sem
InsP_3_*	217^b^	275^b^	355^a,b^	470^a^	59·4
Proportion of InsP_3_ in ΣInsP_3–5_	0·09^b^	0·13^b^	0·19^b^	0·30^a^	0·020
Ins(1,2,4,6)P_4_	ND	ND	ND	ND	
Ins(1,2,3,4)P_4_	750^a^	484^b^	422^b^	284^b^	86·1
Ins(1,2,5,6)P_4_	<LOQ	309^a^	190^b^	318^a^	38·9
Proportion of ΣInsP_4_ in ΣInsP_3–5_	0·34^b^	0·41^a^	0·36^b^	0·39^a,b^	0·015
Ins(1,2,3,4,6)P_5_	365^a^	186^b^	88^b^	ND	43·8
Ins(1,2,3,4,5)P_5_	578^a^	324^b^	436^a,b^	308^b^	63·7
Ins(1,2,4,5,6)P_5_	119^b^	302^a^	96^b^	82^b^	18·8
Ins(1,3,4,5,6)P_5_	75	85	86	85	7·5
Proportion of ΣInsP_5_ in ΣInsP_3–5_	0·57^a^	0·47^b^	0·45^b^	0·31^c^	0·023
InsP_6_	6751^a^	4494^b^	3648^b,c^	2255^c^	544

BD, basal diet; PhyA, BD supplemented with *Aspergillus niger* 3-phytase, Finase^®^ P; PhyE1, BD supplemented with *Escherichia coli* 6-phytase, Quantum^®^; PhyE2, BD supplemented with *E. coli* 6-phytase, Quantum^®^ Blue; InsP_3_, *myo*-inositol trisphosphate; InsP_4_, *myo*-inositol tetrakisphosphate; ND, not detected (the InsP isomer was not detectable in the majority of samples); LOQ, limit of quantification (the InsP isomer was not quantifiable in the majority of samples); InsP_5_, *myo*-inositol pentakisphosphate; InsP_6_, *myo-*inositol 1,2,3,4,5,6-hexakis (dihydrogen phosphate).

^a,b,c^ Mean values in a row with unlike superscript letters were significantly different (*P* ≤ 0·05; Fisher's protected least significant difference test). Mean separation was only computed if the overall *F* test was significant.

* At least one out of the following InsP_3_ isomers: Ins(1,4,5)P_3,_ Ins(1,2,6)P_3,_ Ins(2,4,5)P_3,_ Ins(1,3,4)P_3,_ Ins(1,4,6)P_3_.

In the crop digesta of all phytase-containing treatments, the InsP_5_ pattern was less broad compared with the BD. For the PhyA treatment, the percentage of Ins(1,2,4,5,6)P_5_ in ∑InsP_5_ was significantly higher (*P* < 0·01) whereas the percentage of Ins(1,2,3,4,5)P_5_ in ∑InsP_5_ was significantly lower (*P* < 0·01) compared with the three other treatments (Fig. 1 (A)). In contrast, for PhyE1 and PhyE2, the percentage of Ins(1,2,3,4,5)P_5_ in ∑InsP_5_ was significantly higher, but the percentage of Ins(1,2,4,5,6)P_5_ in ∑InsP_5_ was significantly lower compared with the two other treatments. Ins(1,2,5,6)P_4_ and InsP_3_ also occurred in the crop in high concentrations for the phytase-containing treatments (Table 5). Ins(1,2,5,6)P_4_ appeared for the PhyE1 and PhyE2 treatment in significantly higher concentrations compared with the BD and PhyA (*P* < 0·01) and as the only inositol tetrakisphosphate formed by PhyA. InsP_3_ appeared in significantly higher concentrations for PhyA than PhyE1 and PhyE2 (*P* < 0·01). The proportion of ΣInsP_4_ in ΣInsP_3–5_ was significantly higher for PhyE2 (74 %) than PhyE1 (57 %) and PhyA (14 %) (*P* < 0·01), and that of InsP_3_ in ΣInsP_3–5_ was significantly higher for PhyA (69 %) compared with PhyE1 (15 %) and PhyE2 (13 %) (*P* < 0·01) in the crop. In the proventriculus/gizzard, the InsP_5_ pattern of the phytase-containing treatments was similar to that of the crop except Ins(1,2,3,4,6)P_5_ was not seen in the proventriculus/gizzard (Table 6). Ins(1,2,5,6)P_4_ and InsP_3_ were detected in significantly lower concentrations for PhyA compared with PhyE1 and PhyE2 (*P* < 0·01 for both). A higher proportion of ΣInsP_5_ in ΣInsP_3–5_ and a lower proportion of ΣInsP_4_ in ΣInsP_3–5_ were identified for PhyA compared with PhyE1 and PhyE2 (*P* < 0·01).

In the segments of the small intestine, almost the same InsP isomers were detected in all treatments, except Ins(1,2,5,6)P_4_, which occurred only in the phytase treatments (Tables 7 and 8). In the duodenum/jejunum, Ins(1,2,3,4,6)P_5_ appeared for the first time for PhyA and PhyE2 and again appeared for PhyE1. Ins(1,2,3,4)P_4_ was the predominating InsP_4_ isomer for all treatments. The concentrations of Ins(1,2,3,4,5)P_5_ in the duodenum/jejunum were similar for the BD and the PhyA, but significantly higher for the PhyE1 and PhyE2 treatments (*P* < 0·01) (Table 7). Ins(1,2,4,5,6)P_5_ was detected in significantly higher concentrations for the PhyA compared with the other treatments (*P* < 0·01). For all phytase treatments, a significantly lower proportion of Ins(1,2,3,4,6)P_5_ in ΣInsP_5_ was determined compared with the BD in the duodenum/jejunum (Fig. 1 (B)) and lower ileum (Fig. 1 (C)) (*P* < 0·01). In the lower ileum, the significant difference in Ins(1,2,4,5,6)P_5_ concentrations persisted (*P* < 0·01) whereas the difference in Ins(1,2,3,4,5)P_5_ concentrations lost significance (Table 8). InsP_3_ was not detectable in any of the treatments in the small intestine.

In the caeca, the differences in InsP patterns between the phytase-containing diets and the BD were less distinct than in other sections. Concentrations of Ins(1,2,3,4,6)P_5_ and Ins(1,2,3,4)P_4_ were lower (*P* < 0·01) and concentrations of InsP_3_ (*P* = 0·03) and Ins(1,2,5,6)P_4_ (*P* = 0·04) partially higher when phytases were supplemented, especially for PhyE2 (Table 9). The specific InsP isomers of the supplemented phytases were still present in the caeca. The predominating InsP_5_ isomer was Ins(1,2,3,4,5)P_5_ for all phytase treatments. For the PhyA treatment, significantly higher concentrations of Ins(1,2,4,5,6)P_5_ were measured compared with the other treatments (*P* < 0·01).

## Discussion

### *Myo*-inositol 1,2,3,4,5,6-hexakis (dihydrogen phosphate) hydrolysis and net absorption of phosphorus

In agreement with previous studies that used low-Ca and low-P diets^(^^11^^–^^13^^)^ we found a high rate of InsP_6_ hydrolysis (76 %) and P net absorption (57 %) in the lower ileum. This raises the question of the origin of phytase responsible for this hydrolysis. We found that without supplemented phytase, the majority of InsP_6_ hydrolysis occurred by the end of the duodenum/jejunum, but hydrolysis still continued in the ileum and caeca. In the small intestine of broilers, the greatest endogenous mucosa phytase activity was found in the duodenum^(^^8^^,^^10^^)^. Phytate-degrading activity has also been reported for different lactic acid bacteria isolated from chicken intestine^(^^31^^)^. Thus, intestinal InsP_6_ hydrolysis was the result of a combination of endogenous and microbiota phytase, with as-yet-unknown contributions from each source. When phytase activity in different segments of the digestive tract was compared, the highest activity was found in the caeca^(^^32^^)^ and Kerr *et al.*^(^^14^^)^ detected higher concentrations of InsP_6_ in the caeca of gnotobiotic compared with conventional broilers. In line with this observation, caecal InsP_6_ hydrolysis determined in the present study was greater than 90 %. It should be noted that retrograde movement of digesta and micro-organisms has been described for all segments of the digestive tract in broilers^(^^33^^)^ and it cannot be ruled out that this affected concentrations of InsP isomers, P and Ti anterior to the caeca.

With supplemented phytase, the crop and the proventriculus/gizzard were the main sites of InsP_6_ hydrolysis in the present study. The differences found between the supplemented phytases in these segments might be related to differences in enzyme kinetics, pH or resistance against gastrointestinal proteases. A higher temperature optimum was reported for *E. coli* compared with *Aspergillus* phytases^(^^34^^,^^35^^)^. At approximate body temperature (42°C), *Aspergillus* phytases show an activity of 85 % of the maximum whereas the activity of some *E. coli* phytases is reduced to 60 % of the *in vitro* analysed maximum^(^^34^^)^. Furthermore, *E. coli* phytases are more resistant than *Aspergillus* phytases against pepsin and pancreatin and show a higher activity at pH 3, which is close to the pH in the proventriculus/gizzard^(^^35^^–^^37^^)^. This might explain why the differences between phytases noted in the crop disappeared in the duodenum/jejunum. Moreover, a residual activity of 93 and 60 % has been found for an *E. coli* and an *Aspergillus* phytase, respectively, after incubation in digesta of the proventriculus^(^^35^^)^.

### Appearance of inositol phosphate isomers: basal diet

Past experiments in poultry have focused on the analysis of InsP_6_ and rarely considered clarification of the location of InsP_6_ hydrolysis^(^^14^^,^^32^^,^^38^^)^. A few studies have investigated the sum of InsP_5_, InsP_4_ and InsP_3_ isomers in digesta samples of poultry, without differentiation among positional isomers^(^^39^^–^^41^^)^. The authors are not aware of any published study investigating positional InsP isomers in the digestive tract of broilers, which led us to making this one of our objectives.

When the BD was fed, the dominating InsP_5_ isomers found in the crop were Ins(1,2,4,5,6)P_5_ and Ins(1,2,3,4,5)P_5_. Ins(1,2,3,4,6)P_5_ and Ins(1,3,4,5,6)P_5_ that occurred in concentrations close to the limit of detection may have originated from the diet (Table 1). The changing pattern of InsP_5_ isomers (Fig. 1 (A)) in the crop compared with the diet affirm InsP_6_ hydrolysis in the crop, as does the additional occurrence of InsP_4_. The appearance of Ins(1,2,4,5,6)P_5_ might have been caused by residual intrinsic soyabean phytase, which is a 3-phytase, that withstood exposure to heat in the desolventiser–toaster and was below the limit of detection in the feed. However, this could also have been caused by microbial phytases because 3-phytases are primarily found in fungi (*A. niger*, *A. terreus*, *A. fumigatus*, *Neurospora crassa*), yeasts (*Saccharomyces castellii*, *Saccharomyces cerevisiae*) and bacteria (*Selenomonas ruminantium*, *Selenomonas lacticifex*, *Megasphaera elsdenii*, *Klebsiella terrigena*, *Pantoea agglomerans*, *Pseudomonas syringae*, *Bacillus subtilis*, *Bacillus amyloliquefaciens*). Phytases of plant origin are primarily classified as 4-/6-phytases but 6-phytases were also found for specific bacteria such as *E. coli*, *Peniophora lycii* and *Bifidobacterium pseudocatenulatum*^(^^7^^,^^42^^,^^43^^)^. Therefore, it remains open whether in the present study Ins(1,2,3,4,5)P_5_ was formed by plant or microbiota phytase. Ins(1,2,5,6)P_4_ (perhaps co-eluted with Ins(2,3,4,5)P_4_) measured in the crop could have been formed by 3- or 6-phytases^(^^5^^,^^7^^)^. A formation by other phosphatases which further degraded Ins(1,2,4,5,6)P_5_ or Ins(1,2,3,4,5)P_5_ to Ins(1,2,5,6)P_4_ also is possible.

The fact that there was so little InsP_5_ in the proventriculus/gizzard suggests that when no phytase is added there is very little InsP_6_ hydrolysis in that acid environment. Some remaining intrinsic plant phytase may rapidly be inactivated at low pH values and in the presence of pepsin and pancreatin^(^^44^^–^^47^^)^. The lack of InsP_3–4_ isomers and the dominance of InsP_5_ isomers in the proventriculus/gizzard further indicate that InsP_5_ hydrolysis is less rapidly advanced. However, InsP and their complexes are soluble under acidic conditions and mineral chelates of lower InsPs are more soluble. Hence fast breakdown of InsP_3–4_ also could have happened in the proventriculus/gizzard.

Following passage through the acid phase of the proventriculus/gizzard, a higher accessibility of phytate in the posterior segments can be assumed. In the duodenum/jejunum, a greater range of InsP isomers compared with those found in the stomach was found for the BD treatment, showing intense hydrolysis of InsP_6_ in this segment. This increase probably was the result of substrate-induced InsP_6_ hydrolysis by microbiota or endogenous mucosa phytase. Phytate-induced phytase production was reported for several bacteria^(^^48^^–^^50^^)^. The results of Schlemmer *et al.*^(^^16^^)^ showed substrate dependence of microbiota phytase activity in the colon of pigs. For mucosal phytase, increased activity with dietary phytic acid was also reported in rats^(^^51^^)^. In addition to an alteration of InsP_6_ hydrolysis and diversity of InsP isomers, the InsP pattern changed between the anterior and intestinal segments of the digestive tract (Fig. 1). This change in InsP pattern suggests the involvement of phosphatases of different origin in different segments, with 3- and 6-phytases dominating in the crop and 6- and 5-phytases dominating in the intestinal segments. Ins(1,2,3,4,5)P_5_ can be formed by bacterial 6-phytase of the intestinal microbiota, for example, an *E. coli* 6-phytase. Ins(1,2,3,4)P_4_ first appeared in the duodenum/jejunum and was the dominating InsP_4_ isomer in all intestinal segments when the BD was fed. It might have been a hydrolysis product of a 5-phytase because the majority of phytases continue dephosphorylation adjacent to a free hydroxyl group. Ins(1,2,3,4)P_4_ (perhaps co-eluted with Ins(1,2,3,6)P_4_) was also characterised as a hydrolysis product of a 5-phytase in lily pollen and *Selenomonas ruminantium* subsp. *lactilytica*^(^^52^^,^^53^^)^. However, as Ins(1,2,3,4)P_4_ was also detected as a minor hydrolysis product of specific 6-phytases, it principally could have been formed by both 5- and 6-phytases. In addition, the involvement of other phosphatases which further degraded InsP_5_ cannot be ruled out. Because 5-phytase is described only for *Selenomonas ruminantium* subsp. *lactilytica*^(^^53^^)^, lily pollen and *Bifidobacterium pseudocatenulatum*^(^^43^^)^, its origin in the intestine of broilers was unexpected. Human gut-isolated *Bifidobacterium pseudocatenulatum* initiates InsP_6_ hydrolysis at the C-6 and C-5 position of the *myo*-inositol ring and proceeds via Ins(1,2,3,4)P_4_^(^^43^^)^, but the authors are not aware of any study that found this species of *Bifidobacterium* in broilers.

For the caeca, the broad pattern of InsP isomers is an indication of a highly diverse microbial population likewise producing several phytate-degrading enzymes. From bacteria occurring in the chicken digestive tract, phytate-degrading activity has been described for *Lactobacillus* spp.^(^^31^^)^, *Enterobacter* spp.^(^^54^^)^, *E. coli*^(^^7^^)^, *Klebsiella pneumoniae*^(^^55^^)^, *Bacillus* spp.^(^^56^^)^, *Bifidobacterium* spp.^(^^57^^)^ and *Pseudomonas aeruginosa*^(^^58^^)^. Differences between the InsP pattern of the intestinal segments might be caused by the differing microbial community composition as described by Lu *et al.*^(^^59^^)^ and coupled with the variations in activity of the endogenous phytase. Ins(1,3,4,5,6)P_5_, which appeared in the caeca, indicates the activity of a phosphatase in the caeca that initiates hydrolysis at the C-2 position of the inositol ring. If this was not undegraded Ins(1,3,4,5,6)P_5_ from the feed then this finding contradicts the general assumption that phytate-degrading enzymes are unable to cleave the axial phosphate group of the *myo*-inositol ring.

### Appearance of inositol phosphate isomers: phytase treatments

The second objective of the present study was to investigate the InsP_6_ degradation pattern of different phytase supplements and their effectiveness in releasing phosphate in different segments of the digestive tract. For the PhyA treatment, Ins(1,2,4,5,6)P_5_ was the predominant InsP_5_ isomer whereas Ins(1,2,3,4,5)P_5_ was predominant for the PhyE1 and PhyE2 treatments. This shows, for the first time, that in the crop of broilers the patterns are very similar to the *in vitro* pattern of hydrolysis of these 3- and 6-phytases, initiating InsP_6_ hydrolysis at the d-3 (l-1) and d-6 (l-4) positions of the inositol ring^(^^5^^,^^7^^)^. Ins(1,2,5,6)P_4_ (perhaps co-eluted with Ins(2,3,4,5)P_4_) and InsP_3_, both of which were present in high concentrations for the phytase treatments, are the two other main hydrolysis products of the three phytases. This pattern conforms with *in vitro* results that showed d-Ins(2,3,4,5)P_4_ as a hydrolysis product of *E. coli* and d-Ins(1,2,5,6)P_4_ of *Aspergillus* phytase^(^^5^^,^^7^^)^. The proportion of ΣInsP_4_ in ΣInsP_3–5_ was higher for PhyE1 and PhyE2 compared with PhyA whereas the proportion of InsP_3_ in ΣInsP_3–5_ was higher for PhyA in the crop. Accumulation of *myo*-inositol tris- and bisphosphates following hydrolysis by *A. niger* phytase has already been shown *in vitro*^(^^60^^)^. In contrast, a fast progression from InsP_5_ to InsP_4_ is expected for *E. coli*, but InsP_4_ accumulated. This result corresponds to *in vitro* findings in which InsP_4_ accumulated during InsP_6_ hydrolysis by *E. coli* phytase and was later slowly hydrolysed to InsP_3_^(^^6^^)^. Because the lower-molecular-weight esters of InsPs have a lower mineral-binding strength than InsP_6_ or InsP_5_^(^^61^^)^, the solubility of these esters in the small intestine will be improved, allowing access to them by the endogenous phytase/phosphatases^(^^62^^)^.

In the duodenum/jejunum and to some extent also in the ileum, the InsP_5_ and InsP_4_ isomers specifically formed by the respective supplemented phytases were present in higher concentrations compared with both the BD and the other phytase treatments. Thus, further activity of the enzymes can be assumed in these intestinal segments. However, the proportion of Ins(1,2,3,4,6)P_5_ in ΣInsP_5_ was significantly lower, and concentrations of Ins(1,2,3,4)P_4_ tended to be lower for the phytase treatments compared with the BD treatment in the duodenum/jejunum and lower ileum. Supplementation of phytase tends to reduce lactic acid bacterial count and significantly reduce *E. coli* count in the ileal digesta of broilers^(^^63^^)^, and, as mentioned earlier, both bacterial groups were suspected to be involved in phytate degradation. Aydin *et al.*^(^^63^^)^ speculated that the decrease is related to a possible reduction in the quantity of substrate available to the intestinal microbiota. If there was a reduction of lactic acid bacteria (for example, *Bifidobacteria*) this might explain the decrease in 5-phytase activity for all phytase-containing treatments whereas a reduction of *E. coli* bacteria might explain the decrease in 6-phytase activity for PhyA. Furthermore, a reduction in intestinal mucosal phytase activity has been reported when chickens were supplemented with phytase^(^^10^^)^. Thus, a decrease in activity of endogenous mucosal phytase might also have contributed to different InsP pattern between the phytase and the BD treatments in the small intestine. In the lower ileum, the significant differences between treatments in Ins(1,2,4,5,6)P_5_ concentration persisted, but the differences in Ins(1,2,3,4,5)P_5_ concentration did not. An explanation could be that the activity of supplemented phytases was significantly reduced at this point due to increasing pH and/or proteolytic degradation. Igbasan *et al.*^(^^35^^)^ detected a residual activity of 60 and 55 % for an *Aspergillus* and 87 and 80 % for an *E. coli* phytase in the jejunal and ileal digesta, respectively. Intestinal phosphatases might continue to hydrolyse InsP_3–5_ isomers formed by the supplemented PhyE1 and PhyE2, but InsP_5_ isomers formed by PhyA seemed to be less degradable by intestinal phosphatases. In this regard, Yu *et al.*^(^^64^^)^ showed that Ins(1,2,4,5,6)P_5_ was a more potent aggregator of protein at low pH compared with Ins(1,2,3,4,5)P_5_ which probably would reduce susceptibility of this isomer to phosphatase activity in the intestine.

### Conclusions

We conclude that broilers and their microbiota have a high capacity to hydrolyse InsP_6_ in the intestine. The differentiation between InsP_6_ hydrolysis products of endogenous or microbiota phytases and their contribution to InsP_6_ hydrolysis in different segments still requires experimental work. Phytase supplements are more effective in the anterior than in the intestinal segments of the digestive tract, supporting *in vitro* properties. The main InsP_6_ degradation products of *Aspergillus* and *E. coli* phytases as determined from *in vitro* studies are also formed in the crop and proventriculus/gizzard of broilers. Differences in InsP_6_ hydrolysis between PhyE1 and PhyE2 compared with PhyA existing in the crop disappeared until the ileum. InsP_4_ accumulated in the crop when PhyE1 and PhyE2 were used. However, InsP_3_ accumulated when PhyA was used. It became apparent that the hydrolytic cleavage of the first phosphate group is not the only limiting step in phytate degradation in broilers.
